# FODMAPs—Do they really affect IBS symptoms?

**DOI:** 10.3389/fmed.2023.1123576

**Published:** 2023-03-02

**Authors:** Elise Nordin, Carl Brunius, Rikard Landberg, Per M. Hellström

**Affiliations:** ^1^Department of Biology and Biological Engineering, Food and Nutrition Science, Chalmers University of Technology, Gothenburg, Sweden; ^2^Department of Medical Sciences, Gastroenterology/Hepatology, Uppsala University, Uppsala, Sweden

**Keywords:** irritable bowel syndrome, FODMAPs, double-blind, control, baseline

## Background

Several dietary adaptions have been suggested to successfully reduce irritable bowel syndrome (IBS) symptoms; prebiotics, probiotics, gluten-free diet and low FODMAP (Fermentable Oligosaccharides, Disaccharides, Monosaccharides, and Polyols) diet (1–3). The low FODMAP diet has evolved as the most promising diet therapy (3). Against a theoretical background, the low FODMAP diet is accepted as a treatment strategy in healthcare (4) and efforts are made to implement foods low in FODMAPs on the market (5). However, current evidence for the low FODMAP diet is weak (3): Most reported studies are small and lack double or even single blinding and a majority of the studies have focused on FODMAP eliminations rather than provocations. Trials eliminating FODMAPs from the diet have consistently been shown to reduce IBS symptoms (3). However, removing foods from the diet poses a risk to confound the effect of the intervention with that of placebo, since the blinding is lost. The low FODMAP diet is well known among IBS patients, hence there is a high risk that prior knowledge will shape the clinical responses to a sizable extent, potentially even greater than the actual intervention (6). Another well-known situation is the Hawthorne effect, i.e., change of behavior in response to being observed, which may affect the outcome of the study (6).

## Recent knowledge

Recently our research group performed a double-blind, placebo-controlled, randomized 3-way crossover study with a large number of subjects with IBS (7). After introduction of a diet low in FODMAPs excluding gluten, participants were exposed to week-long provocations with high doses of either FODMAPs and gluten or placebo. The exposure dose was 1.5 times the daily intake for an average person. Despite provocation with such high doses, IBS symptoms were only modestly elevated after the FODMAP intervention, and no effect of the gluten intervention was measured by the IBS severity scoring system (IBS-SSS).

**Figure 1:**
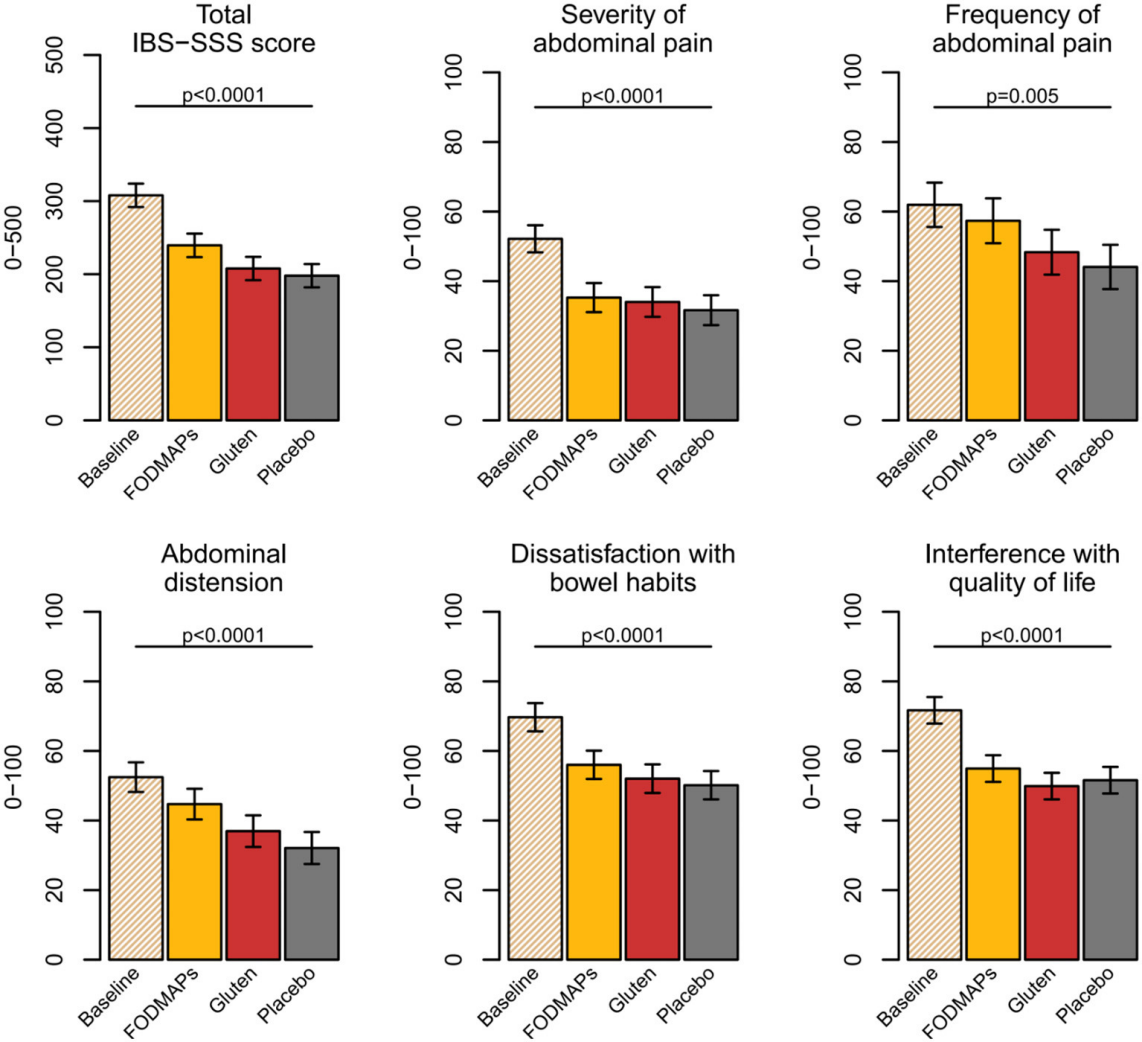
IBS-SSS scores at baseline and after the FODMAPs, gluten, and placebo interventions. Data presented as estimated marginal means, confidence interval (%) and *p*-value from type 3 test. FODMAPs, fermentable oligo-, di-, monosaccharides, and polyols; IBS-SSS, Irritable bowel syndrome Severity Scoring System. This figure from doctoral thesis by Elise Nordin Chalmers, University Technology 2023.

An interesting observation from this trial was that the symptom severity was much worse at the screening visit [mean (SD); 308 ± 50] than after any of the week-long provocations with FODMAPs, gluten or placebo (240 ± 91, 208 ± 91, 198 ± 91) (Figure 1). Our study is not the first to observe this phenomenon. Hustoft et al. (8) found similar results after introducing a low FODMAP diet and thereafter provoking with one specific FODMAP constituent, i.e., fructo-oligosaccharides (FOS). By comparing baseline (freeliving conditions) to a low FODMAP diet during the 3 week run-in period, the authors concluded that a low FODMAP diet was effective since IBS symptoms were drastically reduced. The limited increase in IBS symptoms after the FOS provocation was hypothesized to relate to provoking with one FODMAP component only. However, also in our trial, the effect of FODMAP provocation was minor, even though the FODMAP composition reflected that of the general population but at a higher dose. It seems that symptoms from everyday life, including food habits, by far outweigh symptoms exclusively related to the provocations. Therefore, FODMAPs seem to have a minor effect on IBS symptoms.

A systematic review and metanalysis concluded that a low FODMAP diet reduces IBS symptoms by 45 points on the IBS-SSS scale (9). In our trial, the FODMAP diet challenge increased the IBS-SSS to a similar extent (42 points), suggesting it to be a reasonable estimate. All studies from the systematic review concluded that a low FODMAP diet alleviates gastrointestinal symptoms, with no major discussion about the effect size despite the consensus recommendation that 50 IBS-SSS points are required for a clinical improvement (10). Thus, neither our study, nor the meta-analysis support that FODMAPs have an effect > 50 IBS-SSS units.

## FODMAPs causing symptoms?

Increased awareness is needed considering that the low FODMAP diet has been remarkably effective in comparison to baseline (8, 11, 12). However, in fact, comparison to baseline is strongly discouraged (13). On the other hand, there is only a small or no difference in the effectiveness in reducing IBS symptoms following the low FODMAP diet, general dietary advice, traditional dietary advice, or a gluten-free diet (12, 14–16), hence, diets with a large difference in FODMAP content. In line with these findings, FODMAP provocations have been performed with a large range of doses, from 1.7 to 19 g per day (8, 17, 18) in IBS patients, and 5–20 g FOS per day (19, 20) in healthy, with only mild increases of gastrointestinal symptoms, mainly flatulence, bloating and abdominal pain along with a lack of apparent dose-response. The few available double-blind studies that have included both healthy and people with IBS (21–23), have suggested that FODMAPs cause more severe symptoms in people with IBS, although with some inconsistencies in results: Abdominal pain was higher in IBS subjects compared to healthy, although it did not differ between fructan and control exposure (22). Moreover, those studies were small and employed high doses of fructan. Effect sizes at doses commonly consumed need to be further evaluated. Before drawing conclusions, both people with IBS and healthy need to be studied within the same trial with an adequate control (i.e., not baseline), including evaluation of dose-response.

In light of our findings and the reported, but largely neglected, findings from previous studies (8, 11, 12, 14, 15, 24), we do question the practice of conducting clinical trials to evaluate effects of dietary components such as FODMAPs or gluten in IBS if we cannot assure adequate study conditions. IBS symptoms are clearly related to other factors beyond diet, such as psychological factors (4). Given such factors together with the high placebo response in IBS (25, 26), the importance of a randomized, double-blind, controlled study design has long since been raised (25, 26). In addition, dietary confounding is a major challenge when supplementing foods (6). Therefore, as a basic requirement a double-blind design should substitute the same food(s) in each intervention, effectively ensuring that outcome differences should be purely related to the interventions.

## The complexity of IBS

It is known that IBS is a complex condition, and general guidelines recommend that IBS should be treated from a holistic perspective, integrating medical treatment, lifestyle and dietary adaptations and behavioral therapy (4). Several trials have in fact shown that other interventions can be effective in reducing IBS symptoms, for example acupuncture, cognitive behavioral therapy, hypnotherapy, meditation, and yoga (4), although strong evidence for their efficacy is lacking due to methodological concerns such as lack of blinding (4). Recently, an interesting study (27) concluded that the low FODMAP diet is effective, but at the same time exposure-based cognitive therapy, i.e., the consumption of FODMAPs to target the fear of inducing IBS symptoms is also considered effective (27). A suggested explanation for this was that these two treatments should attract different type of subgroups of people with IBS. However, randomized control trials have shown that both hypnotherapy and yoga were equally effective as a low FODMAP diet (28, 29), indicating that the effectiveness of these different treatment regimen is not related to specific subtypes of IBS.

To conclude, even though elimination of FODMAPs could be part of a holistic IBS treatment, it should be noted that they are complex dietary fibers which are part of a healthy diet and in line with official dietary guidelines (30, 31). This calls for justification based on stronger objective evidence than the theories of saccharide fermentation in IBS that are presently at hand. To gain robust evidence, large double-blind dietary studies with an adequate comparator group are needed. Furthermore, effect size in response to interventions needs to be further discussed and dose-response studies in subjects with and without IBS are highly warranted.

## Author contributions

EN drafted the text. CB, RL, and PH revised the text critically. All authors contributed to the article and approved the submitted version.
